# Fueling the Seed: Growth Factors and Cytokines Driving Cancer Stem Cells in Gynecological Malignancies

**DOI:** 10.3390/ijms262311462

**Published:** 2025-11-26

**Authors:** Alessandro Sarcinella, Juan Sebastian Guerra Villacis, Maria Felice Brizzi

**Affiliations:** Department of Medical Sciences, University of Turin, 10126 Turin, Italy; alessandro.sarcinella@unito.it (A.S.); juansebastian.guerravillacis@unito.it (J.S.G.V.)

**Keywords:** growth factors, cytokines, gynecological cancers, cancer stem cells, tumor microenvironment

## Abstract

Gynecological cancers remain a major global health burden due to their high incidence, molecular heterogeneity, and frequent resistance to conventional therapies. Beyond well-established genetic alterations and targeted treatments, growing attention has been directed toward the role of cancer stem cells (CSCs), a rare tumor subpopulation with self-renewal, differentiation, and tumor-initiating capacities. CSCs are sustained by a specialized microenvironment, the cancer stem cell niche, where growth factors, cytokines, hypoxia, and stromal interactions converge to promote stemness, chemoresistance, and metastatic potential. In breast cancer, signaling axes such as EGFR, IGF, TGFβ, and HGF/c-Met critically regulate CSC expansion, particularly in aggressive subtypes like triple-negative tumors. In ovarian cancer, factors including HGF, VEGFA, IGF, and stromal-derived BMPs drive CSC plasticity and contribute to relapse after platinum therapy. Endometrial CSCs are supported by pathways involving TGFβ, BMP2, and Netrin-4/c-Myc signaling, while in cervical cancer, VEGF, IGF-1, Gremlin-1, and TGFβ-mediated circuits enhance stem-like phenotypes and drug resistance. Cytokine-driven inflammation, especially via IL-3, IL-6, IL-8, IL-10, and CCL5, further fosters CSC survival and immune evasion across gynecologic malignancies. Preclinical studies demonstrate that targeting growth factors and cytokine signaling, through monoclonal antibodies, receptor inhibitors, small molecules, or cytokine modulation, can reduce CSC frequency, restore chemosensitivity, and enhance immunotherapy efficacy. This review highlights the interplay between CSCs, growth factors, and cytokines as central to tumor progression and relapses, emphasizing their translational potential as therapeutic targets in precision oncology for gynecological cancers.

## 1. Introduction

Gynecological cancers, including breast, ovarian, cervical, and endometrial malignancies, represent a major global health challenge, with incidence and mortality strongly influenced by geography, socioeconomic status, and healthcare access [1,2]. Molecularly, recurrent alterations in TP53, PIK3CA, PTEN, BReast CAncer gene 1/2 (BRCA1/2), and DNA mismatch repair genes drive uncontrolled proliferation and genomic instability, with marked heterogeneity in ovarian and endometrial cancers [1]. Risk factors include age, hereditary predisposition, obesity, reproductive history, and persistent HPV infection [2]. Currently, several therapeutic strategies focus on targeting tumor-intrinsic signaling pathways, angiogenesis, homologous recombination deficiency, hormone receptors, and immunologic mechanisms. Corresponding targeted agents include inhibitors of key signaling pathways, anti-angiogenic compounds, poly (ADP-ribose) polymerase (PARP) inhibitors, selective estrogen receptor downregulators (SERDs), and immune checkpoint inhibitors [3,4]. One of the greatest challenges in cancer therapy is the development of tumor resistance to conventional treatments. Consequently, research focus has increasingly shifted toward the cancer stem cell (CSC) population. CSCs represent a small subset of tumor cells characterized by their abilities for self-renewal, differentiation, metastatic potential and therapy resistance [5,6]. This review examines the molecular mechanisms underlying gynecologic cancers, with particular emphasis on the influence of microenvironmental factors, such as growth factors and cytokines, on the expansion and survival of CSCs. The aim is to underscore the therapeutic potential of targeting CSCs as a novel approach within personalized medicine.

## 2. Gynecological Cancers

### 2.1. Breast Cancer

Breast cancer represents the most prevalent malignancy affecting women globally; according to the recent 2022 GLOBOCAN report, female breast cancer accounted for approximately 2.3 million cases worldwide [7]. The primary risk factors associated with breast cancer are female sex and age [8]. Additional factors, such as the age at first childbirth, onset of menopause, pre-existing autoimmune disorders, and lifestyle habits, also contribute to disease susceptibility [9]. Approximately 20–25% of hereditary breast cancer cases harbor germline mutations in the BRCA1 or BRCA2 genes, which are key breast cancer susceptibility loci. Tumors linked to BRCA1 mutations generally exhibit a more aggressive clinical course and are typically classified within the basal-like molecular subtype [10]. Breast cancer represents a highly heterogeneous malignancy, in which three principal biomarkers, the estrogen receptor (ER), progesterone receptor (PR), and human epidermal growth factor receptor 2 (HER2), play a central role in molecular classification and therapeutic strategies guidance [11]. According to the molecular characteristics of tumor cells, breast cancer can be categorized into distinct subtypes, each displaying unique biological and clinical properties, differential therapeutic responses, and variable prognostic outcomes. Breast neoplasms can be divided into Luminal A (ER and/or PR^+^ HER2^−^ and 58.5% prevalence), Luminal B (ER and/or PR^+^ HER2^−^ or HER2^+^ and 14% prevalence), HER2^−^ enriched (ER and/or PR^−^ HER2^+^ and 11.5% prevalence), and Triple Negative (ER^−^, PR^−^ and HER2^−^ and 16% prevalence) [12]. In women with ER^+^/PR^+^ breast cancer, first-line therapy typically involves a taxane-based chemotherapy regimen, with or without anthracyclines [13]. These patients could also receive hormone therapy-based treatment, which aims either to inhibit the release of these hormones or to impair their receptor-mediated activity [14]. Tamoxifen, for instance, acts by competing with endogenous estrogen for binding to its receptor, thereby preventing estrogen–receptor interaction and modulating receptor signaling in a tissue-specific manner [15]. Moreover, hormone therapies also include aromatase inhibitors (AIs), such as Anastrozole and Letrozole, that hamper androgen conversion to estrogens by the enzyme aromatase. Luminal A/B tumors frequently harbor BRCA1 or BRCA2 mutations, which impair DNA repair mechanisms. Under normal conditions, PARP enzymes help resolve single-strand DNA breaks; thus, PARP inhibition results in the accumulation of double-strand breaks, ultimately leading to cancer cell death. PARP inhibitors such as Olaparib and Talazoparib have demonstrated significant survival benefits in patients carrying BRCA mutations [16]. For second-line treatment, options include Capecitabine, Eribulin, Vinorelbine, or previously unused anthracyclines or taxanes to enhance therapeutic outcomes [17]. Cyclin-Dependent Kinase 4 and 6 (CDK4/6) inhibitors combined with endocrine therapy are effective for ER^+^/HER2−metastatic breast cancer, yielding markedly higher response rates compared to endocrine therapy alone. Agents such as Palbociclib, Ribociclib, and Abemaciclib selectively inhibit CDK4/6, thereby interfering with cell cycle progression and preventing uncontrolled tumor cell proliferation [18]. Patients with HER2^+^ breast cancer generally have a worse prognosis and a more aggressive disease than patients whose tumors do not express the membrane protein. HER2 is a transmembrane tyrosine kinase encoded by the *Erbb2* gene, which regulates several key cell processes, including cell growth, migration, and angiogenesis [19]. At present, monoclonal antibodies such as Trastuzumab and Pertuzumab, together with the tyrosine kinase inhibitor (TKI) Lapatinib, represent the main therapeutic options for patients with metastatic breast cancer exhibiting HER2 overexpression [20]. Several anti-HER2 therapies have significantly improved outcomes in both early and advanced HER2^+^ breast cancer [21]. The standard first-line regimens combine Pertuzumab, Trastuzumab, and either Docetaxel or Paclitaxel [22], while Trastuzumab with chemotherapy remains the cornerstone of treatment, greatly enhancing disease-free and overall survival [21,23]. Trastuzumab emtansine (T-DM1) is an antibody–drug conjugate that combines trastuzumab with the cytotoxic agent DM1. It is used as a second-line therapy in patients previously treated with trastuzumab and is also indicated for early-stage HER2^+^ breast cancer [24]. Other targeted agents include Lapatinib, a dual HER1/HER2 tyrosine kinase inhibitor that remains effective when combined with trastuzumab or chemotherapy, despite its higher toxicity profile [23]. Another noteworthy agent is Trastuzumab-Deruxtecan, a next-generation antibody–drug conjugate that couples trastuzumab with the topoisomerase I inhibitor DXd [25]. Additionally, Neratinib, an irreversible inhibitor of HER1, HER2, and HER4, is used after trastuzumab-based therapy to reduce the risk of disease recurrence in selected patients [26]. Triple negative breast cancer (TNBC) is identified as a breast tumor displaying the deficiency of ER, PR, and HER2 [27] with a high immune and stromal infiltration [28]. TNBCs frequently express basal/myoepithelial cytokeratins such as CK5/6, CK14, and CK17, and commonly display epidermal growth factor receptor (EGFR) overexpression and TP53 mutations [28]. This subtype is linked to a poor clinical outcome and high recurrence rate after 5 years from the first diagnosis [29,30,31]. TNBC exhibits a worse clinical course with respect to other breast cancer subtypes and occurs more frequently in patients carrying BRCA1 mutations [22]. Chemotherapy has long represented the standard treatment for TNBC [32], but recent advances have expanded therapeutic options to include PARP inhibitors for patients with BRCA mutations and immunotherapy for tumors expressing Programmed Death-Ligand 1 (PD-L1^+^) [33]. Indeed, for BRCA-mutated TNBC, platinum-based agents such as carboplatin or cisplatin, along with PARP inhibitors, are recommended as first-line treatment, as platinum compounds induce extensive DNA damage and trigger apoptosis [34]. In PD-L1^+^ patients, the combination of Atezolizumab or Pembrolizumab with nab-paclitaxel has demonstrated significant clinical benefit [35]. For BRCA–wild-type and PD-L1–negative tumors, single-agent taxane therapy (paclitaxel or docetaxel) remains the standard approach, whereas combination regimens involving anthracyclines, cyclophosphamide, or platinum agents plus taxanes are recommended for more aggressive disease presentations [36]. Additionally, novel targeted therapies against EGFR, Fibroblast Growth Factor Receptor (FGFR), Vascular Endothelial Growth Factor (VEGFR), and mTOR are under investigation for TNBC treatment [37]. Advances in gene expression profiling have enabled the identification of new molecular subtypes of breast cancer. In 2007, a distinct subtype called claudin-low was described, characterized by reduced expression of tight junction proteins (claudin-3, -4, and -7) and E-cadherin [38]. These triple-negative tumors, observed in both human and murine models, display limited epithelial traits and enhanced stem-like properties [39]. Subsequent studies revealed that the tumor-initiating cells’ (TICs) genomic signature, derived from CD44^high^/CD24^low^ cells and mammospheres, is specifically enriched in claudin-low tumors [40,41]. Moreover, this CD44^high^/CD24^low^/claudin-low profile increases following neoadjuvant chemotherapy or hormone therapy, reinforcing the link between this subtype and stemness-associated, therapy-resistant phenotypes [40]. Claudin-low tumors generally exhibit poor responses to conventional chemotherapy and are associated with shorter relapse-free and overall survival. However, their prognosis largely depends on the intrinsic molecular subtype underlying each tumor [41,42]. Claudin-low tumors display limited sensitivity to chemotherapy and generally have a poor prognosis, indicating that current treatment regimens are often ineffective in managing this subtype [41,42]. However, claudin-low tumors exhibit strong immune cell infiltration [42,43], elevated PD-L1 expression, immunosuppression mediated by regulatory T-cells and a low mutational burden [44], features that may critically influence their response to immunotherapeutic treatments.

### 2.2. Ovarian Cancer

Despite making up only about 3% of all female cancers worldwide, ovarian cancer is still a serious clinical concern because of its aggressive nature and late-stage diagnosis [45]. Indeed, it has the highest death rate (45%) among gynecological cancers and is significantly less common [46]. Age, inherited high-risk syndromes (hereditary breast-ovarian cancer linked to BRCA1/2), Lynch syndrome mutations (MLH1, MSH2), nulliparity, endometriosis, prolonged estrogen exposure and obesity are known risk factors [47]. Based on tumor behavior, molecular characteristics, and histological origin, ovarian cancer is categorized as a heterogeneous illness. The principal histotypes of epithelial ovarian carcinoma are high-grade serous, low-grade serous, endometrioid, clear cell, and mucinous; each of them presents distinct morphological, molecular, and clinical characteristics [48]. High-grade serous carcinoma (HGSC), which accounts for approximately 70% of epithelial ovarian cancers, is characterized by mutations in the TP53 gene and frequent alterations in BRCA1/2 or deficiencies in homologous recombination repair mechanisms. Current evidence supports the hypothesis that serous tubal intraepithelial carcinoma (STIC) located in the distal fallopian tube represents the principal precursor lesion of HGSC [49]. On the other hand, KRAS, BRAF, or NRAS mutations identify low-grade serous carcinoma (LGSC), which develops from benign or borderline serous tumors. Mismatch repair-deficient and POLE-mutated subtypes are identified within the endometrioid group, and endometrioid carcinoma (around 10%) and clear cell carcinoma (between 5 and 10%) are regularly associated with endometriosis and frequently carry ARID1A, PIK3CA, and PTEN mutations [50]. Despite being uncommon (approximately 3 to 4%), mucinous carcinoma is molecularly unique, exhibiting high rates of KRAS mutations, TP53 alterations, and sporadic HER2 (*Erbb2*) amplification [51]. In stage III/IV disease, neoadjuvant chemotherapy is frequently administered prior to primary treatment, which entails complete cytoreduction via primary or interval removing surgery [52,53]. In certain advanced instances, the anti-angiogenetic drug Bevacizumab is added to standard adjuvant therapy, which consists of paclitaxel and carboplatin, to increase progression-free survival, particularly in high-risk patients [54]. PARP inhibitors, such as olaparib, niraparib and rucaparib, are used as maintenance therapy after platinum therapy has been successful [55]. PARP/immunotherapy combinations (Niraparib + Pembrolizumab) produce response rates of approximately 25 to 45% and provide long-lasting disease control [56]. Recently, the FDA approved a novel antibody-drug conjugate, Mirvetuximab-Soravtansine, which targets folate receptor-alpha in platinum-resistant epithelial ovarian cancer, after single trials revealed objective response rates of about 32% [57].

### 2.3. Cervical Cancer

The fourth most prevalent disease in women worldwide, cervical cancer is caused by oncogenic human papillomavirus (HPV) types, especially HPV 16 and 18, which continually infect the cervical epithelium [58]. Around 350,000 people died, and 660,000 new cases were reported globally by the year 2022 [59]. About 80–85% of cervical cancers are squamous cell carcinomas (SCC), with HPV being the primary cause [60]. About 15–25% of cases are adenocarcinomas, which are further classified according to HPV status: HPV-independent variations (like gastric-type, clear cell, mesonephric, and endometrioid adenocarcinomas), HPV-associated usual type, and mucinous subtypes (such as villoglandular, stratified mucin-producing carcinoma, and micropapillary) [61]. Adenosquamous carcinoma, glassy cell carcinoma, small and big cell neuroendocrine carcinomas, and other uncommon histologies such as adenoid basal, mucoepidermoid, and “undifferentiated” carcinoma are further epithelial subtypes [62]. The cornerstone of curative treatment for locally advanced cervical cancer is concurrent chemoradiotherapy with external beam radiation followed by high-dose-rate brachytherapy, which dramatically improves overall survival when compared to radiotherapy alone [63,64]. Immune checkpoint inhibitors have transformed the treatment in the context of recurrent or metastatic disease. Adding pembrolizumab to platinum-based chemotherapy, with or without bevacizumab, increased median overall survival to 24.4 months against 16.5 months [65]. Additionally, the FDA recently approved Cemiplimab, a PD-1 inhibitor, for PD-L1^+^ recurrent/metastatic illness [66]. Tisotumab-Vedotin (Tivdak), an antibody-drug conjugate, has become a unique targeted therapy [67]. Lastly, adoptive cell transfer with tumor-infiltrating lymphocytes (TILs) exhibits promise; phase II results indicate that some patients who received extensive pretreatment experienced full responses [68].

### 2.4. Endometrial Cancer

The most prevalent gynecological cancer, endometrial cancer, arises from the glandular epithelium of the uterine lining. It primarily affects postmenopausal women, whose median age is between 60 and 67 years old [69,70]. Because of enhanced peripheral induction of androgens to estrogen in adipose tissue, which encourages endometrial proliferation and chronic inflammation-mediated DNA damage, obesity continues to be the largest modifiable risk factor [71]. Based on data from the World Cancer Research Fund, there were approximately 420,000 new cases and 98,000 deaths globally in 2022. Estimates for 2025 indicate that there would be approximately 69,000 new cases and 14,000 deaths from the disease, with younger, premenopausal women seeing the highest rates [72]. Endometrioid (EEC) and non-endometrioid (NEEC) tumors are the two main categories of endometrial carcinoma, and each has a unique prognosis and set of molecular characteristics. About 75 to 80% of cases are endometrioid carcinomas, which usually correlate to estrogen-driven, lower-risk disease with glandular anatomy and favorable outcomes, especially with low-grade [73,74]. About 20 to 25% of endometrial cancer cases are NEECs, which are aggressive by nature and are no longer graded but are automatically considered a high risk. These include serous carcinoma, clear cell carcinoma, carcinosarcoma, undifferentiated carcinoma, mixed histologies, and the recently identified mesonephric-like adenocarcinoma. In particular, the poor prognosis of endometrial clear cell carcinoma (ECCC), a rare but clinically relevant cancer, is attributed to its characteristic papillary or tubular development, lack of ER/PR expression, and frequent association with p53 aberrant or high copy number of different molecular profiles [75]. The basis of recent endometrial cancer treatment is surgical intervention, which is followed by precisely individualized adjuvant and systemic medicines based on the patient’s unique characteristics, histological type, and molecular classification. The usual course of treatment for early-stage illness is still total hysterectomy with bilateral salpingo-oophorectomy, frequently combined with lymphadenectomy or sentinel lymph node imaging [76]. Adjuvant chemotherapy with carboplatin and paclitaxel is commonly administered to patients with intermediate- or high-risk histologies, such as serous, clear cell, or high-grade endometrioid carcinomas [77]. This is often performed in conjunction with immunotherapy agents like PD-1 or PD-L1 inhibitors (Dostarlimab or Pembrolizumab), which have been shown to improve progression-free and overall survival in tumors that lack mismatch repair (dMMR) [77]. With response rates of roughly 33% overall and 48% in high microsatellite instability (MSI)/dMMR tumors, Lenvatinib and Pembrolizumab have become a powerful option in the recurrent or advanced setting, especially for mismatch repair-proficient (pMMR) tumors [78]. Additionally, EZH2/EZH1 inhibitors like Tulmimetostat in ARID1A-mutant tumors are presently being assessed in clinical studies [79].

## 3. The CSC Niche

**Figure 1:**
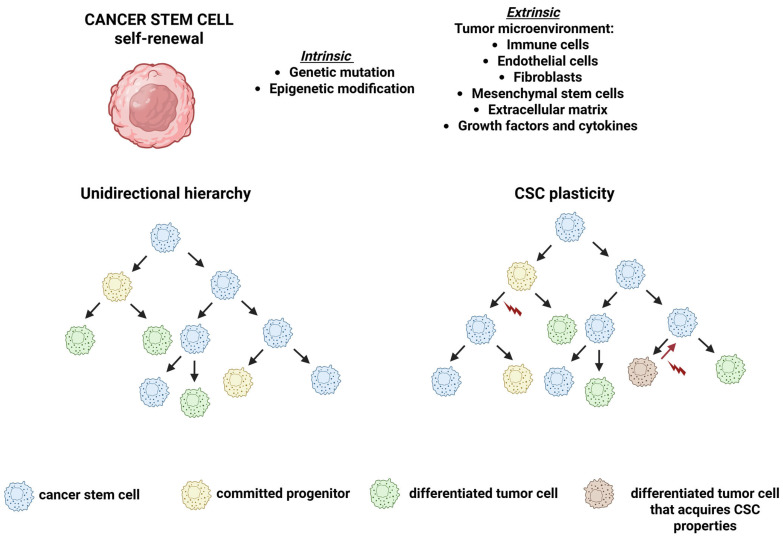
The CSCs Model. The classical CSC model posits a unidirectional hierarchy in which only CSCs can generate the bulk of the tumor through either symmetric division, to self-renew, or asymmetric division, to produce differentiated cellular progeny. In this framework, the hierarchy is rigid, and the notion of progenitor cells reverting to a stem-like state is precluded. However, accumulating evidence indicates that tumor hierarchies are more flexible than initially proposed. The CSC plasticity model suggests that cancer cells can do a bidirectional transition between non-CSC and CSC states. In this context, stemness and CSC plasticity are regulated by a combination of intrinsic and extrinsic factors, which may act independently or cooperatively over time. As a result, non-CSCs can function as a reservoir to replenish CSC populations during tumor progression. In the figure, this process is represented by a lightning bolt, reflecting either microenvironmental stimuli or (epi-)genetic alterations [80,81,82,83]. Created with BioRender.com.

Cancer cells within a single tumor exhibit multiple phenotypic states, each characterized by distinct functional properties. Among this intratumoral heterogeneity, CSCs represent a subpopulation endowed with self-renewal capacity, clonal tumor-initiating potential, and long-term repopulating ability [80,81,82,83]. Over the past decade, the CSC concept has gained increasing prominence. According to the “CSC model” (Figure 1), tumor cell populations are hierarchically organized, with CSCs occupying the apex of this hierarchy. Naturally, CSCs account for less than 5% of the total tumor mass, yet each CSC can initiate a new tumor when transplanted into suitable animal hosts. The CSC model is grounded in the hypothesis that both genetic and non-genetic factors shape a hierarchically structured tumor, in which a self-renewing CSC subpopulation drives the long-term clonal maintenance and expansion of the neoplasm. Because of their tumorigenic potential and capacity to sustain tumor growth, CSCs are also referred to as TICs. Moreover, their ability to give rise to multiple cell lineages mirrors a fundamental property of normal stem cells.

**Figure 2:**
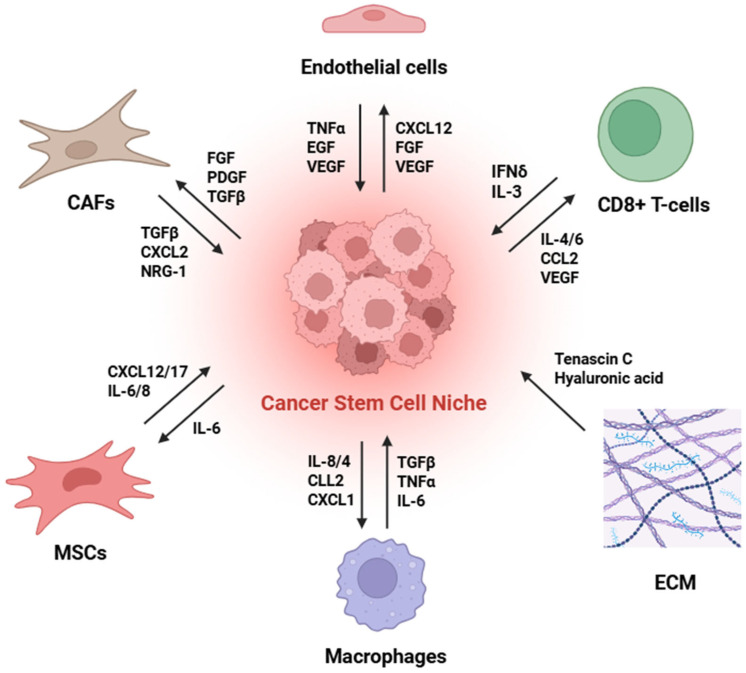
Cancer stem cell niche. The tumor microenvironment (TME) and the intratumoral CSCN play pivotal roles in shaping cancer cell state. Beyond merely hosting tumor cells, the TME exhibits a highly dynamic architecture composed of extracellular matrix (ECM) scaffolds, stromal cells, growth factors, vascular networks, and various immune cell populations. Specific combinations of microenvironmental cues such as inflammation, hypoxia, vascularization, and matrix stiffness can promote stemness and enhance tumorigenic potential. Moreover, different and complex niches may coexist within a single tumor, thereby contributing to the intratumoral heterogeneity [82,87]. Created with BioRender.com.

Furthermore, the identification and isolation of CSCs rely on the presence of characteristic molecular markers. Indeed, among the gynecological malignancies, the most popular surface proteins to isolate and recognize CSCs populations are CD44, CD24, CD133 and CD117. Importantly, these cell subpopulations express high levels of ATP binding cassette (ABC) efflux transporters (especially ABCB1, ABCG2 and ABCC1) and present elevated aldehyde-dehydrogenase (ALDH) activity, which in turn promotes pronounced chemoresistance and persistent self-renewal [4,84,85,86,87]. CSC markers are tumor type-specific and, akin to normal stem cells, are maintained within their specialized microenvironment, referred to as the Cancer Stem Cell Niche (CSCN). The CSCN is a specialized microenvironment in which structural components, soluble factors, and cell–cell crosstalk with neighboring stromal-derived cell types collectively sustain CSC fitness. The CSCN comprises fibroblasts, endothelial and perivascular cells, tissue-associated macrophages, extracellular matrix components, and soluble mediators produced by tumor or stromal cells [88]. A dynamic bidirectional interplay occurs between CSCs and their niche, through which CSCs can modulate the surrounding microenvironment to support their own proliferation, differentiation, invasion, and metastatic potential (Figure 2) [89].

Multiple interconnected microenvironmental factors and adaptive survival mechanisms that CSCs use to maintain stemness and advance tumor growth are what drive the establishment of the CSCN. First, in rapidly growing tumors, oxidative stress and hypoxia stabilize reactive oxygen species (ROS) and hypoxia-inducible factors (HIF-1α, HIF-2α). These factors upregulate genes linked to stemness (OCT4, NANOG, SOX2), activate epithelial-to-mesenchymal transition (EMT) pathways, and induce angiogenic factors, creating a supportive vascularized microenvironment that is specifically designed for CSC maintenance [88,89]. The tumor microenvironment’s chronic inflammation produces high levels of cytokines and chemokines, including IL-6, IL-8, and TNFα, which act through STAT3, NF-κB, and PI3K/Akt signaling to increase the number of CSCs, encourage the dedifferentiation of non-CSC tumor cells through EMT, and recruit immunosuppressive cells [89]. CSCs and stromal cells such as cancer-associated fibroblasts (CAFs), mesenchymal stem cells (MSCs), and endothelial cells engage in reciprocal paracrine crosstalk that results in extracellular matrix (ECM) remodeling and the release of growth factors that promote self-renewal and the development of perivascular niches that serve to physically anchor CSCs and strengthen self-renewal pathways. Essential for CSC homeostasis and plasticity, ECM proteins (hyaluronan, collagen, and laminin) and key adhesion molecules (integrins, E-cadherin, and N-cadherin) promote cell polarity and asymmetric division [88,89]. With all the different factors involved, this allows the CSC niche to maintain stemness and grow in a hierarchical pattern, protect CSCs from immune surveillance and conventional treatments, and use dynamic reciprocal signaling loops to promote tumor growth, metastasis, and recurrence [88,89].

### De-Differentiation: Back to the Future

CSCs possess defining hallmark traits that distinguish them from the tumor cells’ bulk. These include their ability to initiate tumors when transplanted into immunocompromised mice, their capacity for self-renewal, and their potential to generate differentiated progeny representing various tumor lineages [90]. While CSCs were once considered fixed and hierarchically distinct, growing evidence reveals that differentiated cancer cells can reacquire stem-like traits under specific conditions. This cellular plasticity, the ability to revert to a cancer stem cell (CSC) state, is increasingly recognized as an emerging hallmark of stemness [90]. While tumor initiation, self-renewal, and plasticity share overlapping molecular mechanisms, each represents a distinct aspect of CSC biology. Tumor initiation reflects the capacity of CSCs to engraft, adapt, and establish tumors in new environments, whereas self-renewal ensures the long-term maintenance of the CSC pool, sustaining continuous tumor propagation [90]. Together, these hallmarks capture both the adaptability and persistence that underpin CSC-driven tumor growth and recurrence. Growing evidence indicates that differentiated cancer cells can revert to a stem-like state, suggesting that cancer stemness is a dynamic cellular condition rather than a fixed identity [91]. Under certain stimuli, such as stress or microenvironmental factors, signaling pathways governing stemness become reactivated, driving de-differentiation into a progenitor-like phenotype [91]. Cellular plasticity can occur through de-differentiation (reversion to a less mature state), blocked differentiation (maintenance of a progenitor-like state), or trans-differentiation (conversion to a different lineage with distinct characteristics). This adaptability enables cancer cells to reacquire stemness, thereby promoting therapy resistance, metastasis, and tumor relapse [91,92]. Importantly, CSC traits often emerge in only a subset of tumor cells harboring the same driver mutations, underscoring the critical role of the tumor microenvironment. For instance, hepatocyte growth factor (HGF) secreted by cancer-associated fibroblasts (CAFs) can enhance Wnt/β-catenin signaling in neighboring cancer cells, thereby promoting stemness, clonogenicity, and metastatic potential [93,94,95]. Collectively, both cell-intrinsic mechanisms and microenvironmental cues cooperate to induce and sustain CSC states. Studies have demonstrated that factors secreted by cancer-associated fibroblasts (CAFs) can induce stemness across multiple tumor types. In breast cancer, a specific CAF subset has been shown to promote stemness and chemoresistance, being enriched in therapy-resistant tumors and situated in close proximity to ALDH1^+^ CSCs [96]. Co-culture with these CAFs increases the population of ALDH1^+^ and CD44^+^CD24^−^ CSCs, mainly through the secretion of IL-6 and IL-8. Similarly, in ovarian cancer, transforming growth factor β (TGFβ)–activated cancer-associated mesothelial (CAM) cells enhance stemness via CD44 activation [97]. Lineage-tracing analyses revealed that clonogenic CSCs are concentrated at the tumor edge, indicating that tumor growth is driven by spatially defined microenvironmental cues rather than intrinsic CSC markers like LGR5 [98]. Moreover, CAF-derived osteopontin further sustains CSC traits, reinforcing the concept that the tumor microenvironment critically governs CSC functions [98]. Beyond stromal cells, immune and neural components also contribute to CSC conversion. Notably, CD8^+^ T-cells have emerged as unexpected inducers of stemness, as T-cell-derived IFN-γ promotes the expansion of CD44^+^ and CD133^+^ CSCs through FGF2 signaling [99]. Similarly, interferon-γ (IFN-γ) drives the transition of CD90^−^ non-CSCs to CD90^+^ CSCs in breast cancer, underscoring the versatile role of cytokines in modulating stemness [100]. Moreover, macrophages [101,102], neutrophils [103,104], and regulatory T-cells [105,106] dynamically interact with CSCs through direct contact or paracrine signaling, influencing their maintenance and behavior. Additionally, nerve-mediated paracrine signaling has been identified as a key regulator of CSC properties [107,108].

The induction of cancer stemness can also occur independently of the tumor microenvironment. Under stress conditions, cancer cells display remarkable adaptive plasticity, activating stemness programs to survive and acquire a CSC-like state. For example, paclitaxel treatment induces S100A10 expression, which regulates ALDH^+^ CSCs and promotes the transcription of OCT4 and SOX2, driving the acquisition of a CSC phenotype [109]. Various stresses, such as chemotherapy, hypoxia, oxidative stress, nutrient deprivation, and isolation stress, can activate LPAR4-mediated tumor initiation in pancreatic cancer [110]. Similarly, in colorectal cancer, chemotherapy induces a persistent quiescent-like phenotype associated with a fetal stem cell gene signature and enhanced tumor-initiating potential [111].

## 4. CSCs in Gynecological Tumors

### 4.1. Breast CSCs

Although they constitute a minor subpopulation of all cancer cells, breast CSCs (BCSCs) are responsible for cancer progression [112]. Cancer stem cells were first identified in 2003 by Al-Hajj et al., who used the surface markers CD24^(+/high)^ and CD44^(−/low)^ to isolate breast cancer stem cells (BCSCs). The study demonstrated that a small fraction of these cells could generate tumors exhibiting the same heterogeneity as the original tumor when transplanted into immunodeficient mice, whereas other tumor cell populations were unable to initiate tumorigenesis in the same model [113]. In 2007, Ginestier and colleagues identified ALDH1 as a marker of both normal and tumor mammary stem cells, as well as a predictor of poor prognosis [114]. These biomarkers are associated with enhanced growth, adhesion, migration, and invasion potential of BCSCs, which contribute to poor outcomes [115].

Breast cancer stem cells (BCSCs) exhibit distinct marker profiles defined by CD44, CD24, and ALDH expression. The prevalence of CD44^+^/CD24^−^ and ALDH1^+^ BCSCs varies across the four molecular subtypes of breast cancer. Studies indicate that the CD44^+^/CD24^−^ subpopulation is more abundant in luminal A tumors, whereas ALDH1^+^ expression is higher in other subtypes, particularly HER2−positive and TNBCs [116]. This expression pattern correlates with mammosphere formation capacity, as ALDH1^+^ BCSCs significantly generate more mammospheres and show greater tumorigenic potential than CD44^+^/CD24^−^ BCSCs [116]. Moreover, ALDH1 overexpression is linked to increased metastasis, recurrence, and poor prognosis, while CD44^+^/CD24^−^ overexpression shows no significant correlation with these outcomes [117]. A direct correlation between NFκB activation and CD44 expression has also been associated with radioresistance and unfavorable prognosis [118]. Clinical studies indicate that BCSCs are particularly prevalent in TNBC, correlating with high histological grade, estrogen receptor negativity, elevated Ki-67 index, and tumor aggressiveness. Their presence is mainly associated with recurrence, radiation resistance, and metastasis, with CD44^+^/CD24^−^ BCSCs serving as a prognostic marker for metastatic BC [119]. In addition, several additional biomarkers of BCSCs have been identified across diverse breast cancer cell lines, including CD133, ABCG2, SSEA-3, Nectin-4, MUC1, Lgr5, and CD70 [120]. Additionally, certain microRNAs have been identified as markers of BCSC subpopulations, regulating signaling pathways that govern BCSC development and maintenance. Tumor-suppressive microRNAs include Let-7, miR-34, the miR-200 family, miR-30, and miR-600, whereas oncogenic microRNAs include miR-22, miR-155, miR-181, and the miR-221/222 cluster [121]. The foremost signaling pathways that regulate BCSCs include Wnt, Notch, Hedgehog, PI3K/Akt/mTOR, and HER2. Consequently, most therapeutic strategies targeting BCSCs have been designed to inhibit these pathways [122].

### 4.2. Ovarian CSCs

Ovarian CSCs (OCSCs) are major contributors to tumor initiation, progression, and recurrence, driving the pronounced heterogeneity of ovarian cancer. Single-cell RNA sequencing (scRNA-seq) has enabled detailed analysis of OCSC diversity, revealing key stemness genes such as THY1, EPCAM, and CD44 that define distinct subpopulations [123,124,125,126]. The fallopian tube epithelium, especially secretory and intermediate cells, has been identified as a primary source of OCSCs. LGR5, a key stem cell marker in OCSCs and enriched in fallopian tube secretory cells, supports the hypothesis that HGSC arises from LGR5^+^ progenitors [127]. Very small embryonic-like stem cells (VSELs) and germline-derived stem cells have been identified within the ovarian surface epithelium and cortex. These cells express pluripotency markers such as OCT4, NANOG, and SOX2, as well as germline markers including VASA (DDX4) and DPPA3 [128]. The co-expression of CSC markers (CD44, LGR5) with VASA indicates trans-differentiation or dedifferentiation processes contributing to OCSC heterogeneity. OCSCs are controlled by distinct surface markers and transcription factors that maintain their stem-like characteristics. OCSCs are regulated by specific surface markers and transcription factors that preserve their stem-like properties. Key surface markers contribute to tumor growth, metastasis, and chemoresistance. For instance, CD44, a hyaluronic acid receptor, activates PI3K/AKT signaling, thereby promoting cell migration and survival [129]. CD133 enhances tumor initiation and adhesion to metastatic niches, contributing to recurrence [130]. ALDH1 supports detoxification and oxidative stress defense, correlating with platinum resistance and poor prognosis [131,132]. CD24 facilitates immune evasion and spheroid formation, maintaining stemness [133,134]. CD117 (c-KIT) regulates stem cell maintenance and activates MAPK and PI3K/AKT pathways, increasing tumorigenicity [135]. EpCAM promotes adhesion and proliferation, with overexpression linked to metastasis and drug resistance [136]. Collectively, these markers define OCSC stemness and represent potential therapeutic targets against ovarian cancer recurrence [137,138]. Among the transcription factors, SOX2 is a key regulator of OCSCs, as it governs pluripotency, quiescence, and self-renewal, showing higher expression than OCT4 and NANOG in HGSC 3D cultures [139]. SOX2 overexpression in recurrent and treated tumors promotes a quiescent, therapy-resistant state. During this state the tumor is enriched in CD117^+^ and ALDH^+^/CD133^+^ CSCs, marking aggressive, relapse-prone cells [139]. OCT4 maintains stemness and induces EMT by repressing E-cadherin, activating N-cadherin, and stimulating the PI3K/AKT/mTOR pathway, enhancing invasion and drug resistance [140]. NANOG drives EMT and chemoresistance via AMPK/mTOR signaling. KLF4 cooperates with these factors to preserve stemness and increases cisplatin resistance through mTORC1 activation [141].

### 4.3. Cervical CSCs

In cervical cancer, Feng et al. [142] established from cervical tumoral tissue a tumor-derived culture containing stem-like cells capable of self-renewal and forming clonal, nonadherent spheroids. Xenografting as few as 1 × 10^5^ spheroid-derived cells was sufficient to fully recapitulate the original tumor, whereas non-selected cells failed to generate tumors. Cervical cancer stem cells (CCSCs) exhibit distinct molecular and phenotypic profiles that enable their identification, isolation, and functional analysis both in vitro and in vivo. Among the most widely recognized CSC markers, CD44 and CD133, transmembrane glycoproteins expressed across multiple tumor types, are routinely used to isolate CCSCs [143]. Another important marker, CD49f, is highly expressed in CCSCs and particularly useful for their identification. Indeed, sphere-forming cells derived from cervical cancer lines (HeLa, SiHa, CaSki, and C-41) showed an enrichment in CD49f^+^ and CD133^+^ populations compared to adherent monolayers, confirming the stem-like nature of these spheroids [144]. Similarly, a CD49f^+^/ALDH^+^ phenotype associated with the expression of key stemness regulators, including OCT4, NANOG, and β-catenin, has been identified [145]. ALDH^high^ cells displayed stronger tumorigenic potential than ALDH^low^ cells, highlighting ALDH activity as a functional CSC indicator [145]. Further studies linked viral oncogenes to CSC maintenance. Tyagi et al. [146] found that HPV-derived oncoprotein E6 contributes to sustaining CCSC signaling and self-renewal. In both primary tumors and cell line-derived spheroids, E6 overexpression correlated with the presence of stemness-related genes such as OCT4, SOX2, NANOG, LGR1, and CD133 [146]. Among these, SOX2 emerged as a pivotal regulator, as its overexpression in SiHa and HeLa cells enhanced proliferation, colony formation, and tumorigenic potential, whereas differentiated cells lacked SOX2 expression [147]. Nestin, a different stemness-associated protein, was shown to increase sphere-forming efficiency, induce a CD44^high^/CD24^low^ phenotype, similarly to BCSCs, and elevate ALDH, NANOG, and OCT4 levels. These findings suggest that Nestin promotes CSC regulation and cervical cancer progression through its ability to reinforce self-renewal [148]. Similarly, high ALDH1 activity characterizes CCSCs, conferring resistance to cisplatin and correlating with the expression of stem-related transcription factors such as OCT4, NANOG, and KLF4 [149]. Likewise, Cao et al. [150] identified LGR5 as a key regulator of the Wnt/β-catenin pathway in cervical cancer. Overexpression of LGR5 promoted tumorsphere formation, invasion, chemoresistance, and upregulation of stemness markers, including NANOG, OCT4, and KLF4, both in HeLa and SiHa cells [150]. Collectively, these studies indicate that CCSCs are defined by a network of molecular regulators, CD44, CD133, CD49f, ALDH1, LGR5, SOX2, Nestin, and viral E6, that cooperate to sustain self-renewal, tumorigenicity, and therapy resistance. Targeting these pathways could provide an effective strategy to eradicate CSCs and improve cervical cancer treatment outcomes.

### 4.4. Endometrial CSCs

Endometrial cancer exhibits a hierarchical organization with a small subset of cells capable of regenerating tumors resembling the original lesion [151]. These endometrial CSCs (ECSCs) display features such as self-renewal, low proliferation, chemoresistance, and strong tumorigenic potential [151]. ECSCs are typically identified through surface markers, including CD44, CD133, CD117, CD55 and ALDH1, combined with sphere formation and implantation assays to confirm their stemness. CD133^+^ cells in endometrial cancer possess higher proliferative, clonogenic, and migratory capability compared to CD133^−^ cells, along with increased resistance to chemotherapy. These CD133^+^ populations formed spheroids through clonal proliferation, confirming their stem-like behavior [152]. Additionally, CD133^+^ ECSCs from endometrioid adenocarcinoma exhibit high expression of EpCAM, ALDH1, and genes controlling pluripotency [153]. Similarly, ALDH1^high^ cells show upregulation of EMT-related genes and stemness factors (SOX2, NANOG, OCT4, MYC), with ALDH inhibition reducing sphere formation and drug resistance [154]. CD117 (c-Kit), a receptor tyrosine kinase activated by stem cell factor (SCF), also marks ECSCs and promotes proliferation, differentiation, and survival [155]. Furthermore, CD55, a complement regulatory protein, was found to be overexpressed in ECSCs, where it enhances self-renewal and chemoresistance [156]. Inhibition of CD55 using Saracatinib restored cisplatin sensitivity in endometrioid cell lines, indicating its potential as a therapeutic target to overcome drug resistance in endometrial cancer [156]. Hypoxia further enhances ECSC traits by upregulating CD133, ALDH1, SOX2, OCT4, and NANOG, promoting tumor sphere formation [157]. MicroRNAs also modulate ECSC behavior. In particular, miR-423 and miR-135a foster proliferation, invasion, and chemoresistance [158,159], while miR-101 exerts an antagonistic effect, repressing EMT and stemness by targeting TWIST1, ALDH1, and NANOG [160].

## 5. Microenvironmental Cues: Growth Factors and Cytokines Impact on Cancer Stemness

### 5.1. Growth Factors

Growth factors are endogenous signaling proteins that bind to cell surface receptors, primarily receptor tyrosine kinases (RTKs) or serine/threonine kinase receptors, to control vital cellular functions, including proliferation, differentiation, migration, survival, and tissue repair [161]. RTKs like EGFR, VEGFR, FGFR, insulin growth factor receptor (IGF1R), and platelet-derived growth factor receptor (PDGFR) dimerize and auto-phosphorylate upon ligand binding, triggering downstream cascades such as RAS–MAPK, PI3K/AKT/mTOR, PLCγ, and JAK/STAT that incorporate extracellular signals into gene transcription programs governing angiogenesis, apoptosis inhibition, and cell cycle progression [161]. For example, FGF, VEGF, and PDGF frequently work together by co-activating similar downstream pathways, which increases endothelial proliferation and neovascularization in pathological situations like cancer or fibrosis; the FGF-FGFR signaling also plays crucial roles in tissue homeostasis for the tissue repair and possible regeneration after trauma [162]. In a similar manner, IGF-binding proteins and extracellular modulators exercise regulatory control over the IGF1 axis, which interacts with insulin signaling and Wnt/β-catenin pathways to affect metabolic activity, epithelial-to-mesenchymal transition, and mitochondrial biogenesis [163]. TGFβ superfamily ligands, on the other hand, signal through heteromeric serine/threonine kinase receptors, resulting in growth inhibition and apoptosis in normal tissue, or immunosuppression and tumor progression in later oncogenic stages and extracellular matrix deposition [164]. When combined, these growth factor systems create complex linked networks whose feedback control and temporal dynamics dictate cellular reactions ranging from tissue regeneration to cancer [165]. Based on receptor type and ligand structure, growth factors can be systematically categorized into several families. These families are mostly composed of TGFβ superfamily members and RTK ligands. The EGF, FGF, PDGF, VEGF, IGF, colony stimulating factor (CSF), and neurotrophins (NGF, BDNF, NT 3, NT 4, etc.) are prominent families within the RTK class. Each of these families engages distinct receptor subsets, including PDGFR α/β, EGFR/ErbB, IGF1R/IR, VEGFR 1/2/3, FGFR1-4, and Trk/p75NTR, respectively [166,167]. The FGF family is distinguished by its complexity and pleiotropic involvement in development and carcinogenesis. It consists of roughly 22 ligands and four major receptors (with over 48 splice isoforms) [168]. To regulate metabolic homeostasis and mitogenic processes, the IGF family, mainly IGF1 and IGF2, binds to IGF1R and interacts with insulin and Wnt signaling pathways [169]. The TGFβ superfamily, which includes TGFβ isoforms, activins, bone morphogenetic proteins (BMPs), and growth differentiation factors (GDFs), mediates context-dependent effects like immune suppression in pathologic states and growth inhibition in normal epithelial cells versus fibrosis through heteromeric serine/threonine kinase receptors and SMAD proteins [170]. To comprehend the molecular specificity and therapeutic targeting of growth factor signaling in both physiological control and disease situations, this classification system is crucial.

### 5.2. Growth Factor and CSCs Crosstalk in Gynecological Cancer

**Figure 3:**
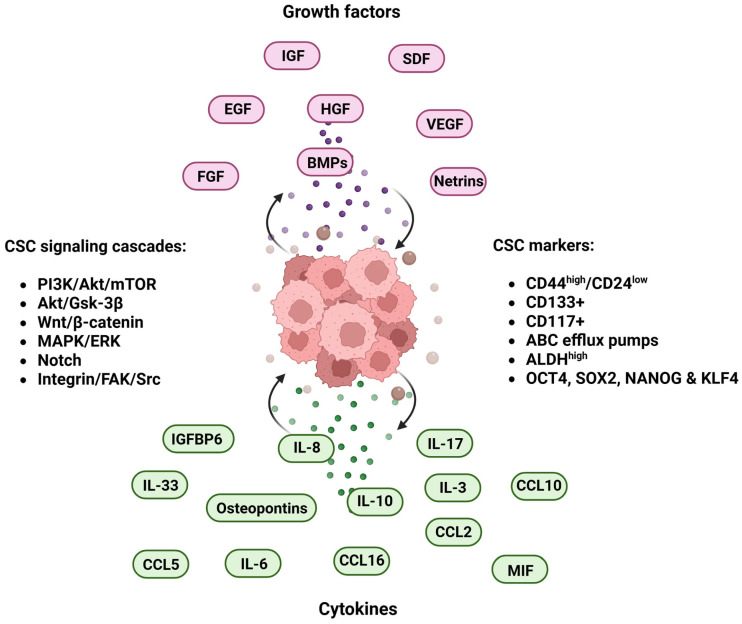
Overview of reviewed growth factors and cytokines involved in CSC regulation in gynecological cancers. Key signaling cascades and surface/intracellular markers are highlighted according to their role in differentiation state. Created with BioRender.com.

Growth factors play a pivotal role in regulating the CSC niche by boosting CSC self-renewal capabilities, de-differentiation switch and resistance to conventional therapies [171] (Figure 3).

In breast cancer, several growth factor signaling pathways are critical for the maintenance of BCSCs [172]. FGF signaling contributes to the enhancement of stemness markers, promotes mammosphere formation, and confers resistance to therapy [173]. In particular, the EGFR axis plays a pivotal role in sustaining CSC populations in TNBC through activation of the PI3K/AKT and MAPK signaling cascades [174]. Additionally, IGF signaling supports CSC functionality by activating and stabilizing the c-Myc oncogene [175]. Crosstalk between adipose-derived stem cells (ASCs) and breast cancer cells, mediated via HGF/c-Met signaling, further regulates tumor self-renewal potential [176]. Additionally, TGFβ promotes stem-like characteristics and chemoresistance in TNBC by modulating the tumorigenic transcription factor RUNX2 [177]. Moreover, BMPs, particularly BMP-4, enhance the expression of BCSC markers and increase sphere formation efficiency through activation of the NOTCH signaling pathway [178]. Most studies on EGFR and FGF signaling in BCSCs rely not only on mammosphere assays and established breast cancer cell lines [172,173,174] but also provide validation in patient-derived xenografts or clinical samples. IGF/IRS2-driven c-Myc stabilization [175] provides a mechanistic insight with promising implications but with limited translational strength. The role of HGF/c-Met crosstalk with ASCs [176] is intriguing for its potential link to recurrence as confirmed from immunostaining in primary breast cancer tissues. Similarly, the TGFβ/RUNX2 [177] and BMP-4/NOTCH [178] axes strongly support stemness in vitro, but evidence of their dominance in patient tumors is scarce. Overall, these findings highlight multiple candidate pathways, but comparative and clinical studies are needed to prioritize therapeutic targets.

In ovarian cancer, HGF activates a specific c-Met/PI3K/Akt signaling axis through a positive feedback loop that promotes cancer stemness and contributes to drug resistance [179]. IGF, abundantly released in ovulatory follicular fluid, enhances stemness and survival of ovarian cancer cells via activation of the IGF-1R/Akt/mTOR and IGF-1R/Akt/Nanog pathways [180]. Notably, carcinoma-associated MSCs (CA-MSCs) within the TME augment the stemness phenotype of ovarian cancer cells through a dysregulated production of BMPs [181]. Moreover, SDF-1, acting through its receptor CXCR4, promotes the proliferation and invasion of CD44^+^/CD117^+^ OCSCs [182]. Furthermore, VEGFA supports stem cell activity in primary human ovarian cancer cultures and cell lines by activating VEGFR2-dependent Src signaling, thereby enhancing tumor sphere formation and increasing ALDH activity [183]. The HGF/c-Met pathway [179] is noteworthy for integrating mechanical forces such as ascitic fluid shear stress, offering a novel translational perspective. The IGF signaling link with follicular fluid [180] provides an elegant explanation of ovulation-associated cancer risk, although large patient cohorts are still lacking. The CA-MSC-driven BMP dysregulation [181] and SDF-1/CXCR4 signaling [182] highlight the importance of the tumor stroma, but their impact on clinical chemoresistance remains underexplored. Among these, the VEGFA/VEGFR2-Src study [183] stands out for using primary patient-derived cultures, strengthening its clinical relevance. Still, comparative studies are needed to determine which pathway predominates in driving OCSC maintenance.

In cervical cancer, members of the TGFβ superfamily promote cancer stem cell-like properties through the activation of the Akt1/Erk1/2/MZF1 signaling axis and upregulation of the stem cell marker CD73 [184,185,186]. Moreover, IGF-1 signaling plays a critical role in promoting chemoresistance and maintaining the stemness of CCSCs, as evidenced by the increased expression of EMT markers and stemness-associated genes such as CD133, OCT4, SOX2, and NANOG. These events resulted in enhanced resistance to cisplatin therapy [187]. VEGF further supports CSC-driven tumorigenesis and invasion via the modulation of miR-146a in tumorspheres derived from HeLa cells [188]. Gremlin-1, a well-known antagonist of BMPs, promotes stemness by increasing the expression of OCT4, NANOG, and SOX2, as well as expanding the ALDH^+^ cell population in cervical cancer [189]. Interestingly, HeLa cells chronically exposed to TGFβ followed by TNFα stimulation exhibit enhanced sphere-forming capacity and self-renewal properties [190]. The TGFβ pathway [184,185,186], the IGF1 signaling axis [187] and the VEGF/miR-146a regulation [188] provide mechanistic insights into chemoresistance but have not been confirmed in primary tumors. Gremlin-1’s role in BMP antagonism [189] represents a promising novel angle with a demonstrated clinical significance about the recurrence event. Future studies should validate these findings using patient-derived organoids or xenografts to confirm their relevance to cervical cancer progression and therapy resistance.

In endometrial cancer, the growth factor-like protein Netrin-4 (NTN4) promotes cancer stemness by activating c-Myc through the integrin β1/FAK/Src signaling pathway [191]. Additionally, stimulation with BMP2 enhances sphere-forming capacity by inducing the expression of the stemness markers c-Kit and CD44 [192]. TGFβ signaling also contributes to ECSC maintenance by regulating the CSC marker CD133 and promoting spheroid formation [193]. Moreover, EGF stimulation of Ishikawa-02 endometrial cancer cells induces SOX2 expression, further supporting the acquisition of a stem-like phenotype [194]. Studies on ECSCs highlight several converging pathways, particularly c-Myc and SOX2 regulation [191,194]. The NTN4/integrin-β1 involvement [191] is relatively novel and provides strong mechanistic evidence, strongly sustained by scRNA-Seq [192] and TGFβ-mediated CSC marker upregulation [193], which is heavily relied upon in multiple stem-related assays, but their prevalence across different endometrial subtypes remains unclear. More work is needed to define whether these pathways act redundantly or hierarchically in maintaining CSC populations, as this could directly influence therapeutic targeting.

### 5.3. Cytokines

Cytokines are little, secreted protein mediators (less than 40 kDa) that coordinate intercellular communication both inside and outside the immune system. They function in autocrine, paracrine, and sometimes endocrine ways to control inflammation, hematopoiesis, immunity, tissue repair, and homeostasis [195]. They are mainly represented by the TNFs, CSFs, IFNs, TGFβ superfamily members, interleukins (ILs), and chemokines and produced by several cell types, such as macrophages, lymphocytes, dendritic cells, and stromal cells [196]. Participation of cytokine receptors creates different signaling cascades, primarily the JAK–STAT pathway but also occasionally NF-κB, MAPK, or SMAD-dependent pathways, which lead to gene transcription programs that decide cell destiny, like survival, proliferation, differentiation, or apoptosis [197]. Depending on concentration, receptor specificity, cellular activation state, and inflammation phase, cytokine interactions can be additive, synergistic, or inhibitory [198]. In terms of function, anti-inflammatory cytokines like TGFβ and IL-10 moderate immune responses and encourage tissue repair and tolerance, while pro-inflammatory cytokines like IL-1β, IL-6, TNF-α, IL-12, and IL-17 stimulate innate immune activation and aid in pathogen removal or chronic inflammatory diseases [199]. Three main criteria are used to classify cytokines: structure, receptor family, and biological function. Regarding their structure characteristics, cytokines are classified into four major superfamilies: the four α helix bundle cytokines (including the IL-2 type, IFN type, and IL-10 type subfamilies, such as IL-2, IL-4, IL-6, IFN-α/β, IFNγ, and IL-10); the IL-1 family (IL-1α/β, IL-18); the IL-17 family; and the cystine knot/TGFβ superfamily [200,201]. Considering the biological function, cytokines are divided into two major groups: pro-inflammatory (like IFNγ, TNFα, IL-12, IL-1β, and IL-17) and anti-inflammatory or regulatory cytokines (like IL-4, IL-10, IL-13, and TGFβ). Activated macrophages, dendritic cells, and T-helper lymphocytes are the main secretors of pro-inflammatory cytokines, which coordinate acute phase reactions, pathogen clearance, leukocyte recruitment, and fever production. Dysregulated overproduction of these mediators is linked to autoimmune diseases, sepsis, and organ failure associated with cytokine dysregulation [202,203]. In contrast, anti-inflammatory cytokines, by suppressing pro-inflammatory cytokine synthesis, downregulate antigen presentation and promote regulatory immune phenotypes. IL-10, TGFβ, IL-1R antagonist (IL-1ra), IL-4, IL-11, and IL-13 act to resolve and suppress inflammatory signaling and preserve tissue integrity and homeostasis [204]. Furthermore, IL-3, produced by TILs [205,206,207,208,209] and tumor-derived endothelial cells (TECs) [210,211], plays a multifaceted role in shaping the TME. By supporting endothelial cell survival and proliferation, as well as promoting the differentiation of hematopoietic progenitor cells, IL-3 enhances tumor angiogenesis and sustains vascular networks necessary for tumor growth [212,213]. Beyond its pro-angiogenic functions, IL-3 exerts a profound influence on immune and stromal cell dynamics within tumors. In TNBC, IL-3 has been implicated in the recruitment of immunosuppressive myeloid cells, the induction of vascular mimicry (VM), and the promotion of EMT, processes that collectively drive tumor progression and metastatic dissemination [214,215]. These findings place IL-3 as a critical mediator of tumor aggressiveness in malignancies characterized by high angiogenic and invasive capability.

### 5.4. Cytokines and CSCs Crosstalk in Gynecological Cancer

Tumor-associated immune cells secrete cytokines that activate key signaling pathways involved in tumor initiation. Additionally, CSCs produce their own attractants, such as chemokines and interleukins, creating a pro-inflammatory microenvironment. This inflamed environment supports the expansion of the CSC subpopulation and promotes tumor progression and metastasis to secondary sites [216] (Figure 3).

In breast cancer, elevated levels of IL-33 have been correlated with increased proliferation, immunosuppression, and upregulation of CSC markers such as CD44 and ALDH1, primarily through activation of the Wnt/β-catenin signaling pathway [217]. In TNBC, M2-polarized tumor-associated macrophages secrete high levels of chemokine (C–C motif) ligand 2 (CCL2), which activates the Akt signaling pathway. This, in turn, enhances the expression and nuclear localization of β-catenin, thereby promoting CSC-like properties [218]. Similarly, polymorphonuclear myeloid-derived suppressor cells (PMN-MDSCs) produce large amounts of C-X-C motif chemokine ligand 2 (CXCL2), which enriches the ALDH^+^ BCSC population via activation of the CXCR2/NOTCH1/HEY1 signaling axis [219]. Additionally, IL-6 and IL-8, produced by cancer cells in an autocrine manner, induce a fibroblastoid morphology and are associated with increased CD44 expression and enhanced self-renewal capacity [220]. Interestingly, IL-10 has been shown to activate the STAT3 pathway, which in turn upregulates the expression and production of CCL16. CCL16 drives the expression of OCT4 and activates the p-AKT/GSK3β pathway, further enhancing CSC traits including ALDH^+^ subpopulation enrichment, chemoresistance, and sphere-forming ability [221]. Furthermore, IL-3, which can also be secreted by breast cancer cells [222], promotes the upregulation of the oncogenic miR-155-5p, thereby impairing the function of the β-catenin destruction complex. This cascade results in sustained c-Myc expression, driving continuous enrichment of CSCs and contributing to enhanced mammosphere formation, expansion of the CD44^high^/CD24^low^ subpopulation, elevated ALDH expression and activity, and increased resistance to doxorubicin. Notably, tumorspheres secrete higher levels of IL-3 than bulk cultures, suggesting the existence of an IL-3–mediated autocrine loop that sustains self-renewal [222]. The IL-33/Wnt [217] and CCL2/β-catenin [218] studies are strong in mechanistic design, but clinical correlation remains limited. The interactions between cells and cytokines within the immune microenvironment represent additional layers of complexity not fully addressed in these works. The CXCL2/CXCR2/NOTCH1 axis [219] adds novelty by connecting myeloid-derived suppressor cells to CSC regulation, contributing to the discovery of a relevant chemoresistance signature. The autocrine loop of IL-6/IL-8 [220] and IL-3 [222] is well documented and provides evidence about the distinct response among different breast cancer subtypes. The IL-10/CCL16/STAT3 study [221] is compelling in showing a cascade that enhances multiple CSC traits using tissue microarrays, making it one of the more promising avenues for translational targeting.

In ovarian cancer, ascitic fluid serves as a valuable resource for assessing the presence of OCSCs and inflammatory mediators [223]. A recent study reported that two distinct stem-like subpopulations, CD44^+^/CD133^−^ and CD44^−^/CD133^+^, positively correlate with IL-10 levels in ascites and VEGF levels in serum [223]. In HGSC, IL-6 regulates stemness features of OCSCs driven by ALDH1A1 expression and activity [224]. Moreover, platinum-based chemotherapy stimulates IL-6 secretion by CAFs, promoting OCSC enrichment in residual tumors following treatment [224]. Elevated levels of pro-tumorigenic cytokines IL-10 and IL-6 have also been reported in CSC/M2-macrophage co-cultured heterospheroids, enhancing both chemoresistance and invasiveness in 3D culture systems [225]. Among the diverse cytokines implicated in stemness, IL-8 secreted by M2-polarized macrophages induces a stem-like phenotype in OCSCs [225]. Furthermore, You et al. [226] demonstrated that CCL5 is highly expressed in OCSCs compared to non-CSCs, and its receptor, CCR5, is upregulated on regulatory T cells (Tregs) in ovarian cancer patients. This CCL5–CCR5 axis facilitates the recruitment of Tregs by OCSCs, contributing significantly to immunosuppression. In addition, it has been shown that T-regs cultured in conditioned medium from CD133^+^ OCSCs ovarian cancer cells exhibited higher IL-10 expression than those subjected to CM from CD133^−^ cells, suggesting that CSC-rich environments enhance the immunosuppressive function of Tregs [226]. In addition, IL-17, a pro-inflammatory cytokine primarily produced by Th17 cells and macrophages, promotes the growth and sphere-forming ability of CD133^+^ OCSCs in a dose-dependent manner [227]. Interestingly, osteopontin, secreted by cancer-associated mesothelial cells, has been shown to enhance chemoresistance and stemness in ovarian cancer by activating CD44 receptors, stimulating PI3K/AKT signaling, and increasing ABC transporter-mediated drug efflux [228]. The correlation between cytokine levels in ascites/serum and CSC subpopulations [223] provides valuable clinical context, but causality is not firmly established. The IL-6/ALDH1A1 axis [224] is among the most robustly characterized mechanisms of OCSC maintenance and chemoresistance, with the novel finding that chemotherapy itself induces IL-6 release being particularly relevant. However, validation in large patient cohorts is needed. The CCL5–CCR5 axis [226] offers a strong immunological link between OCSCs and T-reg recruitment, though functional in vivo evidence remains limited. IL-17 [227] and osteopontin [97] findings expand the landscape of pro-stemness signals but still lack comprehensive validation in clinical specimens. Overall, while the preclinical data are compelling, translational and clinical studies are essential to determine which cytokines are dominant drivers in clinical settings.

In cervical cancer the interplay between CSCs and cytokines remains less well-characterized compared to other gynecological malignancies. However, ASC-secreted factors have been shown to induce IL-6 production and upregulate pluripotency-associated genes such as OCT4, KLF4, and ABCG in HeLa cells [228]. These findings suggest that the adipose tissue microenvironment can enhance the stem-like phenotype of cervical cancer cells. Interestingly, Sato et al. replicated the stemness features of CCSCs by reprogramming human induced pluripotent stem cells (iPSCs) into induced reserve cell-like cells (iRCs). This subpopulation of cells secretes significantly higher levels of inflammatory cytokines, including macrophage migration inhibitory factor (MIF), soluble intercellular adhesion molecule-1 (sICAM-1), and CXCL10 compared to normal cervical epithelial cells [229]. The ASC study [228] highlights the potential role of stromal cells in enhancing CSC traits, but its reliance on HeLa cells limits its translational strength. The iRC model developed from iPSCs [229] is innovative and provides a new experimental platform to study cytokine–CSC interactions, yet its direct relevance to patient tumors remains uncertain. The literature on cytokines in cervical CSCs is still sparse, underscoring the need for patient-derived and clinical data to establish the true impact of inflammatory signaling in this malignancy.

In endometrial cancer a subpopulation of endometrial cancer-derived MSCs (EmCaMSCs) has recently been isolated from patient samples. These cells represent approximately 1–5% of the total tumor cell population and are considered part of the CSC niche [230]. Notably, EmCaMSCs secrete IL-8 and insulin-like growth factor-binding protein 6 (IGFBP6), both of which contribute to immune evasion by suppressing the proliferation of peripheral blood mononuclear cells (PBMCs) [230]. These findings underscore the role of CSC-derived cytokines in promoting tumor-associated immunosuppression. Furthermore, CD133^+^ cells isolated from endometrioid adenocarcinomas, which are resistant to cisplatin and paclitaxel, display a distinct gene expression profile characterized by elevated levels of IL-8. This highlights the critical role of IL-8 in maintaining cancer stemness and chemoresistance in endometrial cancer [231]. The isolation of EmCaMSCs from patient-derived tumors [230] adds important clinical weight to the evidence, confirming that CSC–cytokine interactions are not merely in vitro artifacts. The role of IL-8 as both an immunosuppressive and chemoresistance-promoting factor [152,230] is especially compelling, but mechanistic studies in vivo remain scarce. The consistent association of IL-8 with stemness across multiple cancer types suggests it may be a convergent cytokine pathway, making it a strong candidate for translational therapeutic exploration.

## 6. Growth Factors and Cytokines as Potential Targets for CSC Therapy

### 6.1. Growth Factor-Targeted Therapies

In breast cancer, a fully humanized, high-affinity monoclonal antibody (aNRP2-10) has recently been developed to specifically block the interaction between VEGF and its co-receptor neuropilin-2 (NRP2). Remarkably, aNRP2-10 promotes the differentiation of CSCs into a more chemosensitive and less metastatic phenotype [231]. Notably, the therapeutic is capable of driving CSC differentiation into a more therapy-responsive phenotype with reduced metastatic potential [231]. The development of the aNRP2-10 antibody is particularly noteworthy: it not only disrupts the well-established pro-tumorigenic VEGF/NRP2 axis but also induces a functional phenotypic shift toward a more differentiated state that is more susceptible to chemotherapy, an especially significant outcome given the central role of CSCs in therapeutic resistance. Nevertheless, as a newly developed molecule, its long-term in vivo effectiveness, systemic tolerability, and potential off-target effects remain to be fully elucidated, particularly in light of the broad physiological functions attributed to NRP2. In a different study, Zhang et al. [232] identified a lead compound, YH677, characterized by a tetrahydro-β-carboline scaffold, which inhibits CSC expansion by modulating the TGFβ/Smad signaling pathway, resulting in a dose-dependent downregulation of stem cell markers. The identification of YH677 further expands the therapeutic landscape, showing that TGFβ/Smad pathway modulation can restrain CSC expansion. A clear strength relies on the dose-dependent downregulation of stem cell markers. However, targeting such a pleiotropic signaling cascade raises concerns about specificity and the potential for unexpected adverse effects associated with small-molecule inhibitors. Importantly, EGFR inhibitors have been shown to enhance the efficacy of doxorubicin in BCSC-derived xenograft tumors [233]. The process of VM in BCSCs is mediated by the EGF/Hsp27 signaling axis, inhibition of EGFR phosphorylation via Gefitinib or knockdown of EGFR through lentiviral shRNA effectively abolished in vitro VM activity in BCSCs [234]. Findings on EGFR inhibitors, both their ability to enhance chemotherapy efficacy and to disrupt VM, reinforce the idea that interfering with key signaling hubs shared by tumor growth and CSC plasticity can yield multifaceted benefits. Nonetheless, the clinical translatability of these observations is limited by the heterogeneous expression of EGFR in breast cancer and the common emergence of resistance, underscoring the need for robust predictive biomarkers and more advanced preclinical models.

In ovarian cancer, Poziotinib, a pan-HER inhibitor, significantly reduces sphere formation, viability, and proliferation of OCSCs by disrupting downstream signaling pathways including Wnt/β-catenin, NOTCH, and Hedgehog [235]. Poziotinib’s broad inhibition of the HER family and its downstream effects on several key signaling pathways underscore the therapeutic potential of multi-target inhibitors in disrupting the complex signaling networks that sustain OCSC survival and plasticity. However, its pan-HER activity may also translate into higher toxicity and reduced specificity, raising questions about its tolerability in clinical settings. Inhibition of VEGFR3 preferentially affects CD133^+^ OCSCs, leading to the downregulation of BRCA1 and BRCA2, and thereby restoring chemosensitivity in previously resistant ovarian cancer cell lines [236]. The preferential sensitivity of CD133^+^ OCSCs to VEGFR3 inhibition, together with the associated downregulation of BRCA1/2 and restoration of chemosensitivity, represents an intriguing mechanistic link. While promising, the perturbation of BRCA-related pathways may also carry unforeseen implications for genomic stability, necessitating careful and comprehensive evaluation. Furthermore, the small-molecule AZD4547, a potent FGFR inhibitor, impairs spheroid formation and self-renewal of OCSCs, while also exhibiting anti-angiogenic properties and suppressing in vivo tumor growth in mouse models [237]. AZD4547 adds further support to the relevance of receptor tyrosine kinase (RTK) signaling in maintaining OCSC properties. Its dual impact on self-renewal and angiogenesis is a clear strength, yet FGFR pathway redundancy and compensatory signaling, common in ovarian cancer, may limit its long-term efficacy unless combined with additional targeted agents. Moreover, TGFβ signaling inhibition leads to a significant reduction in self-renewal capacity, tumorigenicity in vitro, and migratory and invasive behavior of OCSCs [238]. Despite these encouraging results, TGFβ’s context-dependent role, as both tumor suppressor and promoter, complicates therapeutic targeting and may lead to unpredictable effects depending on disease stage and tumor microenvironment.

In cervical cancer, inhibition of EGFR phosphorylation leads to downregulation of the stemness marker SOX2 and a reduction in the CSC population in CaSki and HeLa cell lines [239]. EGFR phosphorylation provides a solid mechanistic link between receptor signaling and stemness regulation. However, most data derive from established cell lines such as CaSki and HeLa, which may not fully recapitulate the heterogeneity and microenvironmental influences present in a patient’s tumors. Similarly, Erlotinib, an EGFR-TKI, effectively prevents CSC enrichment in paclitaxel-resistant cervical cancer cells by suppressing IL-6 production [240]. Erlotinib’s action highlights an important intersection between inflammatory signaling and therapy-induced stemness. This underscores a key strength of the strategy: the targeting of feedback loops that CSCs exploit during chemoresistance. Yet, EGFR-TKIs are known for variable efficacy across cervical cancer subtypes, and compensatory cytokine pathways may limit long-term effects. Notably, the VEGF inhibitor Bevacizumab significantly suppresses the enrichment of ALDH^+^ CSCs and enhances the sensitivity of cervical carcinoma to cisplatin therapy [241]. The use of bevacizumab to inhibit VEGF-induced enrichment of ALDH^+^ CSCs and to restore cisplatin sensitivity highlights the relevance of the interplay between CSC and angiogenesis. Moreover, the clinical availability of bevacizumab represents a practical advantage for translational application. In endometrial cancer, delivery of miR-326 encapsulated in superparamagnetic iron oxide nanoparticles (miR-326@SPION) significantly inhibits proliferation and invasion of ECSCs in vitro, as well as tumorigenicity and neovascularization in vivo by targeting the VEGF signaling pathway [242]. A major strength of this approach is the targeted delivery offered by SPIONs, which can improve miRNA stability and cellular uptake. However, nanocarrier-based therapies still face challenges related to biodistribution, long-term safety, and potential off-target effects, and their translation to clinical practice remains limited. Moreover, Shang et al. proposed a novel therapeutic approach by combining EGFR inhibition with NOTCH pathway targeting in CD133^+^ cells, aiming to enhance the efficacy of EGFR inhibitors in endometrial cancer [243]. The combined targeting of EGFR and NOTCH signaling in CD133^+^ cells proposed by Shang et al. offers a compelling strategy to overcome the limited efficacy of EGFR inhibitors in endometrial cancer. By addressing pathway redundancy and the compensatory activation that frequently weakens monotherapies, this dual-inhibition approach highlights the importance of tackling CSC-associated signaling networks in parallel. Nevertheless, the inhibition of two major pathways simultaneously may increase toxicity or disrupt normal stem cell function.

### 6.2. Cytokine-Targeted Therapies

In breast cancer, IL1R2 has been identified as a promoter of TICs self-renewal and tumor progression, especially in TNBC. IL1R2 blockade significantly reduces the BCSCs population, inhibits macrophage recruitment and M2 polarization, and prevents CD8^+^ T-cell exhaustion, collectively contributing to tumor suppression [244]. The major strength of IL1R2 targeting lies in its multi-level impact: reducing BCSC frequency, limiting pro-tumorigenic macrophage recruitment and M2 polarization, and preventing CD8^+^ T-cell exhaustion. This comprehensive modulation of both intrinsic CSC properties and extrinsic immune dynamics is promising. However, the pleiotropic role of IL-1 signaling raises concerns about systemic immune effects, and the extent to which IL1R2 blockade may interfere with normal inflammatory responses remains to be fully elucidated. Notably, IL-24, also known as melanoma differentiation-associated gene-7 (mda-7), selectively reduces BCSCs proliferation without affecting normal mammary stem cells. It impairs BCSCs self-renewal by suppressing the Wnt/β-catenin signaling pathway [245]. The selective activity of IL-24 against BCSCs without harming normal mammary stem cells is another notable advantage, suggesting a favorable therapeutic window. This targeted therapy demonstrated high efficacy and safety in a clinical trial [246].

In ovarian cancer, OCSCs can activate NF-κB and STAT3 signaling through autocrine secretion of CCL5, which promotes their differentiation into endothelial-like cells [247]. Both anti-CCL5 antibodies and CCL5-shRNA have been shown to inhibit endothelial differentiation and tube formation of OCSCs in vitro and in vivo [247]. These phenomena underscore the importance of self-reinforcing inflammatory loops in sustaining CSC phenotypes and promoting VM. Targeting CCL5 with neutralizing antibodies or shRNA demonstrates a clear functional impact by suppressing endothelial differentiation and tube formation, marking CCL5 as a promising therapeutic target. Nevertheless, chemokines often form redundant networks, and compensatory signaling through related ligands may limit the long-term efficacy of CCL5 inhibition. Additionally, signaling via the CXCL12–CXCR4 axis plays a key role in OCSC development. In vivo administration of an oncolytic vaccinia virus (OVV) engineered to express a CXCR4 antagonist effectively reduces metastatic spread and enhances OCSC eradication [248]. The role of CXCL12–CXCR4 signaling in OCSC maintenance further highlights the relevance of chemokine-driven stemness. The use of an oncolytic vaccinia virus engineered to deliver a CXCR4 antagonist represents an innovative therapeutic strategy, combining direct oncolysis with microenvironmental reprogramming. A key strength of this approach relies on its dual mechanism of action disrupting CSC-supportive signaling while simultaneously inducing tumor cell lysis. However, the safety, specificity, and immune effects of engineered oncolytic viruses remain significant translational challenges, particularly regarding viral persistence and host antiviral responses.

In cervical cancer, CSCs enriched in the ALDH^high^ subpopulation are more sensitive to IFNγ-mediated CD8^+^ T-cell killing with respect to ALDH^low^ cells [249]. The increased sensitivity of ALDH^high^ CSCs to IFNγ-mediated CD8^+^ T-cell cytotoxicity contradicts the common assumption that CSCs are generally more immune-evasive. This finding suggests that specific CSC subsets may retain vulnerabilities that can be therapeutically exploited. Interestingly, phenethyl isothiocyanate (PEITC), an inhibitor of tumor necrosis factor-related apoptosis-inducing ligand (TRAIL) signaling, reduces the proliferation and sphere-forming capacity of the CD44^high^/CD24^low^ cell fraction [250]. By suppressing TRAIL signaling—traditionally associated with pro-apoptotic effects—PEITC reduces proliferation and sphere formation, indicating that TRAIL may have context-dependent, pro-survival functions in certain CSC subsets. While this highlights a potential therapeutic window, the pleiotropic nature of PEITC and its broad impact on cellular redox states raise concerns about specificity and off-target effects.

In endometrial cancer, endometrium-derived MSCs (eMSCs) have been shown to suppress both the sphere-forming ability and stemness-related gene expression of endometrial cancer cells. This effect is mediated through secretion of DKK1, which inhibits the Wnt/β-catenin signaling pathway [251]. The observation that eMSCs can suppress sphere formation and downregulate stemness-associated genes through DKK1 secretion is particularly intriguing, as it challenges the prevailing notion that MSCs typically support tumor progression. However, MSCs are highly heterogeneous, and their effects can vary dramatically depending on their source, differentiation status, and environmental cues, raising concerns about reproducibility and clinical applicability. Notably, targeted inhibition of interleukin-6 receptor (IL-6R), which is highly expressed in ALDH^high^ ECSCs, leads to a marked reduction in tumor cell growth, highlighting the critical role of IL-6 signaling in CSC maintenance [252]. The finding that IL-6R is highly expressed in ALDH^high^ ECSCs and that its inhibition significantly reduces tumor growth underscores the importance of inflammatory cytokine signaling in sustaining CSC properties. This provides a strong rationale for targeting IL-6/IL-6R signaling as part of CSC-directed therapies. Yet, IL-6 is a pleiotropic cytokine involved in numerous physiological processes, and systemic blockade may lead to adverse immune or metabolic effects.

## 7. Future Perspectives

Future research on growth factors and cytokine-mediated regulation of CSCs in gynecological malignancies should aim to move beyond pathway-level descriptions toward integrated, clinically actionable models. Single-cell and spatial transcriptomic technologies are expected to redefine CSC heterogeneity, revealing microanatomical niches and context-specific signaling circuits that cannot be captured by bulk analyses. Moreover, deciphering the temporal dynamics of growth factor and cytokine signaling during therapy, particularly during chemotherapy-induced CSC enrichment, will be essential to understanding adaptive resistance. Another priority is the functional validation of candidate pathways in patient-derived organoids, ex vivo tumor slices, and immunocompetent models that better recapitulate stromal, immune, and mechanical cues shaping stemness. Therapeutically, the next generation of CSC-targeted approaches will likely rely on multi-node inhibition, simultaneously blocking redundant RTK and cytokine cascades, or on differentiation-inducing strategies that render CSCs more vulnerable to standard treatments. Combining these agents with immunotherapies and understanding how CSCs manipulate immune subsets through cytokines may open new avenues to overcome immune evasion. Nonetheless, the main limitation of these combinatorial strategies is the considerable burden of anticipated adverse effects. Finally, the integration of CSC-focused biomarkers with multi-omic profiling in clinical trials will be essential for identifying patients most likely to benefit from therapies targeting growth factors and cytokine networks. This strategy can improve patient stratification, provide mechanistic insights into treatment response, and help predict both therapeutic efficacy and potential resistance mechanisms in gynecologic cancers.

## Data Availability

No new data were created or analyzed in this study. Data sharing is not applicable to this article.
